# A predictive ambulance dispatch algorithm to the scene of a motor vehicle crash: the search for optimal over and under triage rates

**DOI:** 10.1186/s12873-022-00609-5

**Published:** 2022-05-06

**Authors:** Ellen Ceklic, Hideo Tohira, Stephen Ball, Elizabeth Brown, Deon Brink, Paul Bailey, Rudolph Brits, Judith Finn

**Affiliations:** 1 Prehospital, Resuscitation and Emergency Care Research Unit (PRECRU), School of Nursing, Curtin University, GPO Box U1987, Perth, WA 6845 Australia; 2 Emergency Medicine, Medical School, The University of Western Australia, Perth, Australia; 3 St John Western Australia, Belmont, WA Australia; 4 School of Public Health and Preventive Medicine, Monash University, Melbourne, VIC Australia

**Keywords:** Ambulance, Dispatch, Lights, Sirens, Motor vehicle crash

## Abstract

**Background:**

Calls for emergency medical assistance at the scene of a motor vehicle crash (MVC) substantially contribute to the demand on ambulance services. Triage by emergency medical dispatch systems is therefore important, to ensure the right care is provided to the right patient, in the right amount of time. A lights and sirens (L&S) response is the highest priority ambulance response, also known as a priority one or hot response. In this context, over triage is defined as dispatching an ambulance with lights and sirens (L&S) to a low acuity MVC and under triage is not dispatching an ambulance with L&S to those who require urgent medical care. We explored the potential for crash characteristics to be used during emergency ambulance calls to identify those MVCs that required a L&S response.

**Methods:**

We conducted a retrospective cohort study using ambulance and police data from 2014 to 2016. The predictor variables were crash characteristics (e.g. road surface), and Medical Priority Dispatch System (MPDS) dispatch codes. The outcome variable was the need for a L&S ambulance response. A Chi-square Automatic Interaction Detector technique was used to develop decision trees, with over/under triage rates determined for each tree. The model with an under/over triage rate closest to that prescribed by the American College of Surgeons Committee on Trauma (ACS COT) will be deemed to be the best model (under triage rate of ≤ 5% and over triage rate of between 25–35%.

**Results:**

The decision tree with a 2.7% under triage rate was closest to that specified by the ACS COT, had as predictors—MPDS codes, trapped, vulnerable road user, anyone aged 75 + , day of the week, single versus multiple vehicles, airbag deployment, atmosphere, surface, lighting and accident type. This model had an over triage rate of 84.8%.

**Conclusions:**

We were able to derive a model with a reasonable under triage rate, however this model also had a high over triage rate. Individual EMS may apply the findings here to their own jurisdictions when dispatching to the scene of a MVC.

**Supplementary Information:**

The online version contains supplementary material available at 10.1186/s12873-022-00609-5.

## Background

Calls for emergency ambulance assistance for motor vehicle crash (MVCs) patients substantially contribute to the demand on emergency medical services [1]. Triage by emergency medical systems (EMSs) is therefore important to ensure the right care is provided to the right patient, in the right amount of time [2]. EMS must determine the priority of the ambulance response. The highest priority (usually where it is recognised there is an immediate risk of death to one or more of the patients at the scene), is where lights and sirens (L&S) are used on the way to the scene. In this setting, over triage can be defined as dispatching an ambulance using L&S to a low acuity MVC. Conversely, under triage involves dispatching an ambulance not using L&S to a MVC, where patients are at immediate risk of death. Under triage is a concern because of the risk of death, or another adverse patient outcome should there be a delay in the arrival of an ambulance on-scene [3]. Over triage, in the context of limited EMS resources, could result in ambulances not being available for other, more time-critical patients as well as the additional risk of an ambulance crashing [4].

An ideal system would match patient need with ambulance dispatch priority. With MVCs people on the scene are usually not medically trained and cannot provide reliable information about medical need. Therefore, there are various methods used by EMS for prioritizing ambulance dispatch to MVCs. Some use codes assigned through a systemized dispatch system, such as the Medical Dispatch Priority System (MPDS); which uses criteria related to the number of resources needed (such as for multi-vehicle crashes), the potential for danger (such as for those involving hazardous chemicals) or those involving high mechanisms of injury (such as rollovers) [5]. However, these codes have been found to have poor predictive ability to identify those patients who require a L&S ambulance response to the scene of a crash [6, 7]. An alternative is to use additional information and characteristics of the MVC that laypersons can easily report at the scene and therefore able to be derived through a set of questions prompted by the dispatcher within the EMS during the call for emergency ambulance services. These characteristics could include road features, speed zone or how many vehicles were involved. While it is not a novel idea that crash characteristics be used to predict injury severity [8, 9], there is a scarcity of research exploring whether crash characteristics could improve the dispatch accuracy in identifying those MVCs that do/do not require an ambulance L&S response to the scene.

### Aim

To develop an algorithm to identify cases for which a L&S ambulance response is required, using MVC characteristics.

## Methods

A population-based retrospective cohort study was conducted on all MVCs attended by St John Western Australia (SJ-WA) ambulance paramedics in the Perth metropolitan area, Western Australia, from 1^st^ Jan 2014 to 31^st^ Dec 2016. Perth had a population of approximately 2 million people and covers an area of 6,400 square kilometres [10]. The road environment is built-up/urban with mandated speeds ranging from 50 km/h in residential areas to 110 km/h on motorways [11]. SJ-WA is the sole, contracted provider of single-tier advanced life support EMS in Perth.

### Data sources

We used two data sources in this study (1) SJ-WA ambulance data and (2) Main Roads Western Australia (MRWA) crash data. The ambulance data comprised information collected during the emergency phone call, as logged through a computer-aided dispatch system (CAD) using the Medical Priority Dispatch System (MPDS) (v. 12) [12]​, and electronic patient care records (ePCRs) entered by paramedics. This includes event date and time, geographical coordinates of the location of the MVC, dispatch code, main problem for which an ambulance was requested (as determined by paramedics), dispatch priority to the scene, priority from the scene to a hospital, patient vital signs, interventions provided (e.g., medications, splinting) and patient disposition (left at scene, died at scene, or transported to hospital).

The study cohort was defined as those MVCs where an ambulance was dispatched as a Traffic/Transportation incident (MPDS Protocol 29), and where paramedics coded the incident as Motor Vehicle Accident. This was to exclude incidents that did not include a motor vehicle (such as single bicycle crashes, as those involving an aircraft or train) within the Traffic/Transportation MPDS category.

The MRWA crash data is a composite dataset of information collated from Western Australian Police (who attended all fatal and critical injury crashes); and drivers involved in the crash. Data includes information pertaining to the crash (such as location, weather and road environment details), as well as information to do with the vehicle (such as make, model, year of manufacturing and safety features) and persons involved (age, sex, role in the crash and injury severity). We limited records in the crash data to those defined as a reportable road crash [13], with crashes that result in damage costing < $5,000 AUD or not resulting in injury, not being required to be legally reported.

### Data linkage

**Fig. 1:**
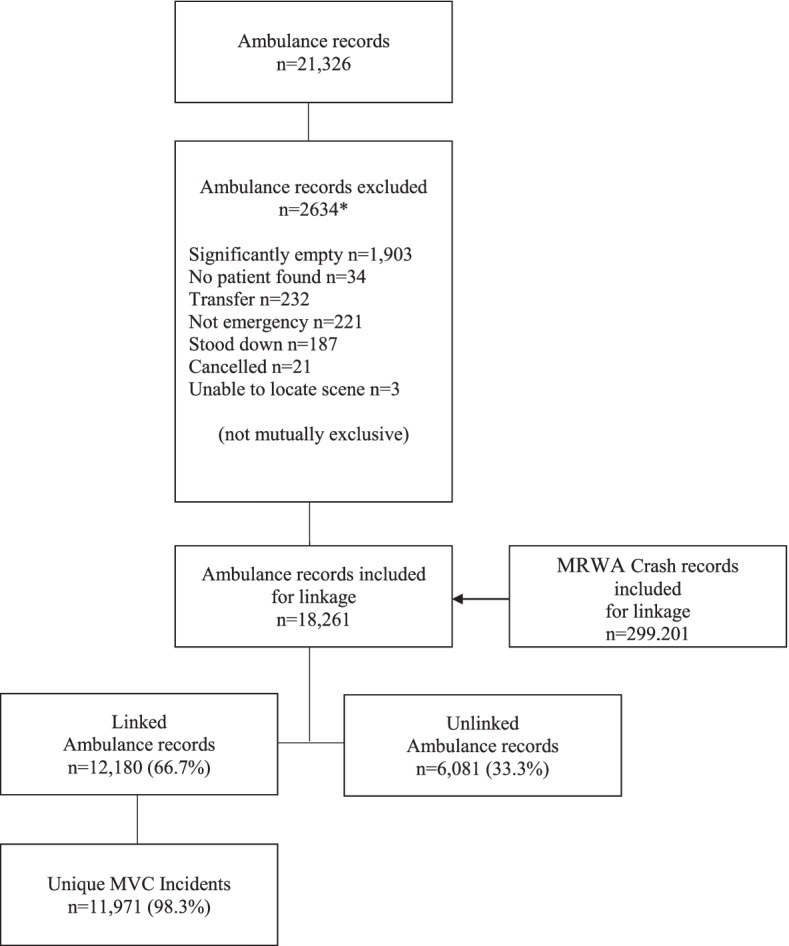
Flow diagram of linkage process between ambulance and motor vehicle crash records.*Significantly empty: no electronic record of assessment, intervention, or clinical case notes;. No patient found: Paramedics arrived on-scene but there was no patient present (e.g. patient absconded); Patient transfer between hospitals; Not emergency: Use of the ambulance for other than delivering patient care, such as transport of equipment; Stood down: Ambulance dispatched but then received a higher priority job or another crew was closer to the scene; Cancelled: Individual calls from the scene and states they no longer require the ambulance; Unable to locate scene: Ambulance is sent but cannot locate the MVC

We linked the ambulance study cohort (defined above) to the MRWA crash data using first and last name, sex, date of birth and vehicle registration number. We used Fine-Grained Records Integration and Linkage Tool (version 2, Emory University, US) and SAS (version 9.4. SAS Institute Inc., Cary, NC, USA) for this purpose. Crash records without a corresponding ambulance record were excluded from the final dataset. The linkage rate was 66.7%, representing the proportion of ambulance records with a corresponding crash record. See Fig. 1. It was not expected that there would be an exact match between the datasets as not all people with ambulance care after a MVC report their crash to MRWA or involve Police.

### Predictor variables

Predictor variables were defined as characteristics of the crash, derived from either the ambulance or crash data, and that bystanders could reasonably describe at the scene, and be coded by EMS. See Table 1 for a full list of the variables included.

**Table 1:** Description of decision tree variables

Variable name	Variable type	Brief description
Accident type	Dichotomous	Intersection/Midblock
Airbag deployed	Dichotomous	Any airbag deployed/No deployed
Anyone ejected	Dichotomous	Anyone ejected (incl. partial)/Everyone not ejected
Anyone not ambulant	Dichotomous	Anyone not ambulant/Everyone ambulant (able to walk)
Anyone trapped	Dichotomous	Anyone trapped in a vehicle/Everyone not trapped
Atmosphere	Nominal	Smoke, Clear, Overcast, Raining, Fog
Child	Dichotomous	Anyone aged ≤ 12 years/Everyone aged ≥ 13 years
Day of the week	Nominal	Day of the week Sunday = 1 etc
Lighting	Nominal	Daylight/Dawn or dusk/Dark with lights on/off/not provided
MPDS dispatch code	Nominal	Medical Priority Dispatch System dispatch codes for Protocol 29
Older	Dichotomous	Anyone aged ≥ 75 years/Everyone aged ≤ 74 years
Raining	Dichotomous	Raining/Clear
Road alignment	Dichotomous	Curved/Straight
Road grade	Nominal	Level/Crest of hill/Slope
Road surface	Dichotomous	Sealed/Unsealed
Rollover	Dichotomous	Any vehicle rolled over/No vehicle rolled over
Single v. Multi-vehicle	Dichotomous	Single vehicle/2 or more vehicles
Speed limit	Ordinal	Posted speed limit (km/h)
Time of day	Continuous	hh:mm
Traffic control	Nominal	Traffic lights/Stop sign/ Give way sign/Zebra crossing/ Railway crossing/ School crossing/No signal or control
Type of intersection	Nominal	4-way/3-way (T-junction)/Roundabout/Bridge/Rail Crossing/Driveway
Vulnerable road user	Dichotomous	Involved vulnerable road user (cyclist, motorcyclist or pedestrian)/No vulnerable road user involved

MPDS dispatch codes for the Traffic/Transportation Chief Complaint (Protocol 29) that were routinely assigned during each call were also included as predictor variables. MPDS dispatch codes are automatically assigned (through the use of the ProQA software) immediately following a set of scripted questions from the emergency medical dispatcher to the caller at the scene. MPDS dispatch codes are those that best describe the incident, such as the code for rollover (D2p), HAZMAT (D3) or sinking vehicle (D2s) [5]. These MPDS dispatch codes were ‘forced’ as the first variable in some of the models to more closely reflect ‘usual state’ should an EMS continue to use the established MPDS.

### Outcome variables

The outcome variable was the need for a L&S ambulance response to the scene of a crash. In SJ-WA a L&S response is the highest priority ambulance response, where L&S are used on the way to the scene. This is also termed a priority one or hot response in some jurisdictions. In SJ-WA there is an operationally defined time to arrive within 15 min for 90% of L&S responses and L&S are not permitted to be used for lower priority responses, such as priority two, three or four. In this study, a L&S response was a binary: yes/no. A MVC was retrospectively determined to have potentially required a L&S response for the following conditions:

- The ambulance priority from the scene to an emergency department was L&S; or.

- Anyone was dead on-scene or in transit; or.

- Anyone had one or more L&S dispatch indicators (described below).

The L&S dispatch indicators were developed by the SJ-WA Clinical Governance Department. The indicators included specific clinical interventions, administered medications and patient clinical observations recorded by paramedics (see Supplementary material).

### Statistical analysis

We analysed data using the Chi-square Automatic Interaction Detector (CHAID) [14] technique for branching using SPSS (version 26. IBM Corp. Armonk, NY,). This decision tree technique was chosen as it allowed for a multi-way split on variables (as opposed to a binary split using a CART technique) and both categorical and numerical variables can be used in the tree.

A CHAID technique splits data into groups based on the relationship between the predictor variables (in our case, MPDS code ± on-scene crash characteristics) and the outcome variable (whether the crash was classified as having required a L&S response). To minimize error rates, misclassification costs were used to penalize incorrectly classified cases. Both the levels and misclassification costs were varied to identify the best decision tree model. Misclassification cost ratios were incrementally increased from 1 until the under triage rates were at 0%, which represents assigning all MVCs as L&S. Misclassification costs allowed us to preference those MVCs that required a L&S response over those that did not. Model A was set to include only MPDS dispatch codes (and therefore could have only a depth of one). Model B included MPDS dispatch codes at the first level and any combination of crash characteristics as other levels. For this model, levels were limited to three. Model C included only crash characteristic and levels were limited to three. Model D included MPDS dispatch codes as the first level and any combination of crash variables, with levels as unlimited. Lastly model D could include any crash variables with unlimited levels.

Over triage rate was defined as the proportion of crashes where a L&S response was determined to have not been required among those for which a decision tree predicted L&S was required (i.e. 1 – positive predictive value). Under triage rate was defined as the proportion of MVCs where a L&S response was determined to have been required, among crashes where the decision tree predicted that L&S was not required (i.e. 1 – negative predictive value). The model with an under/over triage rate closest to that prescribed by the American College of Surgeons Committee on Trauma (ACS COT) was deemed to be the best model: that being an under triage rate of 5% or below and an over triage rate of between 25–35% [15].

### Ethics

Curtin University Human Research Ethics Committee granted approval for this study, as a sub-study of the Western Australia Pre-hospital Record Linkage Project (HR 128/2013). A data licensing agreement was signed with Main Roads Western Australia for use of the crash data. SJ-WA Research Governance Committee gave approval to conduct the study using ambulance data.

## Results

There were 11,971 MVCs attended by SJ-WA emergency medical ambulances in Perth WA during the three years to the 31^st^ of December 2016. Of ambulance records, 66.7% had a matching crash record. (see Fig. 1).

Within the study cohort, the following MPDS dispatch codes has the highest proportion of MVCs attended: 29B1-injuries (19.5%), 29B4 – unknown status/other codes not applicable (18.7%) and 29D2l – High mechanism, vehicle v. bicycle/motorcyclist (9.6%). See Table 2.

**Table 2:** Motor vehicle crash incidents by medical priority dispatch Code (MPDS) and lights and sirens ambulance (L&S) response

**MPDS dispatch code**		**Not L&S**	**L&S**	**Total**	**Total**
	**Brief descriptor**	**n**	**n**	**n**	**Col %**
29D1	Major incident	7	1	8	0.1%
29D1V	Major incident, multiple patients	2	0	2	0.0%
29D1b	Major incident—bus	5	1	6	0.1%
29D1d	Major incident—train	1	2	3	0.0%
29D1f	Major incident—multiple vehicle (≥ 10) pile-up	4	0	4	0.0%
29D2	High mechanism	257	78	335	2.8%
29D2k	High mechanism—all-terrain/snowmobile	2	0	2	0.0%
29D2l	High mechanism- vehicle v. bicycle/motorcycle	905	247	1,152	9.6%
29D2m	High mechanism—vehicle v. pedestrian	672	169	841	7.0%
29D2n	High mechanism—ejection	80	34	114	1.0%
29D2p	High mechanism—rollovers	377	67	444	3.7%
29D2r	High mechanism—possible death at scene	0	1	1	0.0%
29D3	HAZMAT	67	10	77	0.6%
29D3U	HAZMAT, unknown number of patients	3	1	4	0.0%
29D3V	HAZMAT, multiple patients	19	3	22	0.2%
29D3X	HAZMAT, unknown number of patients and additional response required	1	0	1	0.0%
29D3Y	HAZMAT, multiple patients and additional response required	3	0	3	0.0%
29D4	Trapped victim	338	126	464	3.9%
29D4U	Trapped victim, unknown number of patients	59	23	82	0.7%
29D4V	Trapped victim, multiple patients	124	64	188	1.6%
29D4X	Trapped victim, unknown number of patients and additional response required	3	2	5	0.0%
29D4Y	Trapped victim, multiple patients and additional response required	28	21	49	0.4%
29D4n	Trapped victim, ejection	3	0	3	0.0%
29D5	Not alert	325	153	478	4.0%
29D5U	Not alert, unknown number of patients	5	2	7	0.1%
29D5V	Not alert, multiple patients	47	21	68	0.6%
29D5X	Not alert, unknown number of patients and additional response required	0	1	1	0.0%
29D5Y	Not alert, multiple patients and additional response required	3	2	5	0.0%
29D5m	Not alert, vehicle v. pedestrian	0	2	2	0.0%
29D5n	Not alert, ejection	2	4	6	0.1%
29B1	Injuries	2,098	237	2,335	19.5%
29B1U	Injuries, unknown number of patients	41	8	49	0.4%
29B1V	Injuries, multiple patients	470	56	526	4.4%
29B1X	Injuries, unknown number of patients and additional response required	6	1	7	0.1%
29B1Y	Injuries, multiple patients and additional response required	57	5	62	0.5%
29B2	Serious haemorrhage	126	31	157	1.3%
29B2V	Serious haemorrhage, multiple patients	23	3	26	0.2%
29B2X	Serious haemorrhage, unknown number of patients and additional response required	1	0	1	0.0%
29B2Y	Serious haemorrhage, multiple patients and additional response required	5	3	8	0.1%
29B3	Other hazards	589	76	665	5.6%
29B3U	Other hazards, unknown number of patients	71	8	79	0.7%
29B3V	Other hazards, multiple patients	214	29	243	2.0%
29B3X	Other hazards, unknown number of patients and additional response required	6	2	8	0.1%
29B3Y	Other hazards, multiple patients and additional response required	33	4	37	0.3%
29B4	Unknown status/Other codes not applicable	2,054	185	2,239	18.7%
29B4U	Unknown status/Other codes not applicable, unknown number of patients	429	36	465	3.9%
29B4V	Unknown status/Other codes not applicable, multiple patients	442	37	479	4.0%
29B4X	Unknown status/Other codes not applicable, unknown number of patients and additional response required	29	4	33	0.3%
29B4Y	Unknown status/Other codes not applicable, multiple patients and additional response required	31	5	36	0.3%
29A1	1st party caller with injury to not dangerous body area	16	0	16	0.1%
29A1V	1st party caller with injury to not dangerous body area, multiple patients	1	0	1	0.0%
29O1	No injuries (confirmed)	116	6	122	1.0%
**Total**		**10,200**	**1,771**	**11,971**	**100.0%**

For crash characteristics the categories of no one ejected (99.0%), everyone ambulant (97.9%) and no vehicle rolled (96.3%) had the highest proportion of MVCs attended. See Table 3.

**Table 3:** Motor vehicle crash incidents, by crash characteristics and lights and sirens (L&S) response

	**Not L&S**	**L&S**	**Total**	**Total**
	**n**	**n**	**n**	**Col %**
**Accident Type**				
Intersection	4,230	653	4,883	40.8%
Midblock	2,513	586	3,099	25.9%
**Airbag deployed**				
Any airbag deployed	3,280	571	3,851	32.2%
No airbag deployed	6,920	1,200	8,120	67.8%
**Alignment**				
Curve	861	206	1,067	8.9%
Straight	4,669	850	5,519	46.1%
**Atmosphere**				
Clear	3,207	694	3,901	32.6%
Dust/Smoke	5	1	6	0.1%
Fog/Mist	12	3	15	0.1%
Fog/smoke/dust	0	1	1	0.0%
Overcast	277	90	367	3.1%
Raining	358	55	413	3.5%
**Day of the week**				
Monday	1,384	189	1,573	13.1%
Tuesday	1,481	261	1,742	14.6%
Wednesday	1,537	234	1,771	14.8%
Thursday	1,552	271	1,823	15.2%
Friday	1,683	297	1,980	16.5%
Saturday	1,360	258	1,618	13.5%
Sunday	1,203	261	1,464	12.2%
**Ejected**				
Anyone ejected	63	62	125	1.0%
No one ejected	10,137	1,709	11,846	99.0%
**Grade**				
Crest of hill	78	18	96	0.8%
Level	3,321	668	3,989	33.3%
Slope	666	186	852	7.1%
**Lighting**				
Daylight	4,867	824	5,691	47.5%
Dawn/Dusk	479	75	554	4.6%
Dark—street lights on	1,095	257	1,352	11.3%
Dark—Street Lights Off	19	6	25	0.2%
Dark—Street Lights Not Provided	69	46	115	1.0%
**Not ambulant**				
Anyone not ambulant	140	116	256	2.1%
Everyone ambulant	10,060	1,655	11,715	97.9%
**Older**				
Any aged ≥ 75 years	739	120	859	7.2%
Everyone aged ≤ 74 years	9,461	1,651	11,112	92.8%
**Single versus Multi-vehicle**				
2 or more vehicles	5,314	689	6,003	50.1%
Single vehicle	1,423	326	1,749	14.6%
**Surface**				
Sealed	3,321	668	3,989	33.3%
Unsealed	78	18	96	0.8%
**Rollover**				
Any vehicle rolled	377	67	44	0.4%
No vehicle rolled	9,823	1,704	11,527	96.3%
**Trapped**				
Anyone trapped	444	303	747	6.2%
No one trapped	9,756	1,468	11,224	93.8%
**Under**				
Anyone ≤ 12 years	455	92	547	4.6%
Everyone ≥ 13 years	9,745	1,679	11,424	95.4%
**Vulnerable**				
Motor vehicle occupant	6,866	854	7,720	64.5%
Vulnerable	1,943	536	2,479	20.7%
** Total**	**10,200**	**1,771**	**11,971**	**100.0%**

^*^Components may not sum to the total due to missing data

**Fig. 2:**
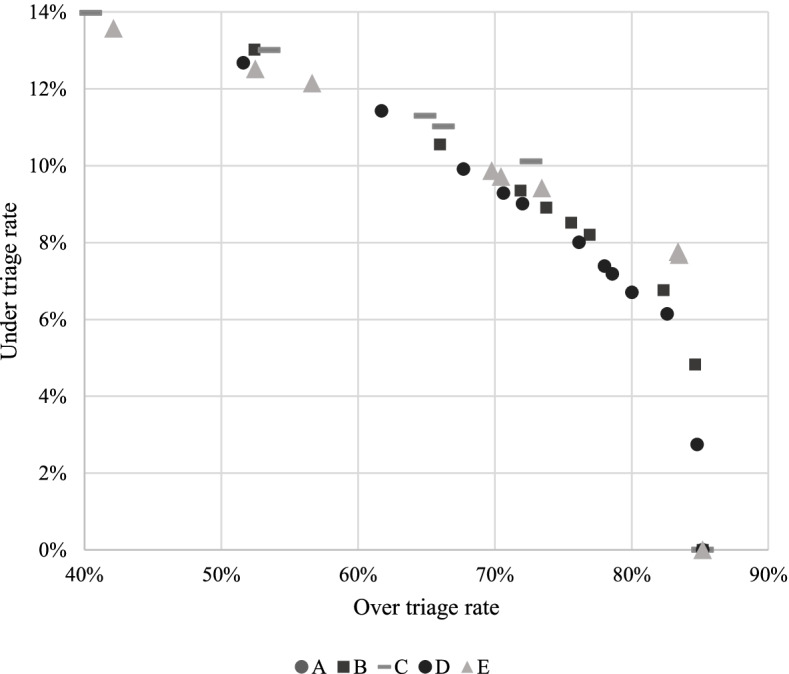
Over/under triage rates of various decision trees. + Each marker represents a different CHAID decision tree, as listed in Table 4. *Model A variables were: MPDS dispatch codes. Model B variables were: MPDS dispatch codes, anyone trapped, vulnerable road user, airbag deployed, atmosphere, road surface. Model C variables were anyone trapped, vulnerable road user, anyone not ambulant, atmosphere, accident type. Model D variables were: MPDS dispatch codes, anyone trapped, vulnerable road user, anyone aged ≥ 75 years, day of the week, single v. multi-vehicle, airbag deployed, atmosphere, road surface, lighting, accident type. Model E variables were: MPDS dispatch codes, anyone trapped, vulnerable road user, anyone aged ≥ 75 years, day of the week, single v. multi-vehicle, airbag deployed, atmosphere, road surface, lighting, accident type

As shown in Table 4, under triage rates ranged from 0% (where all incidents were dispatched at L&S) to 14.1%, in a decision tree with three levels using MPDS dispatch codes along with anyone trapped, vulnerable road use, airbag deployed, atmosphere and road surface. See Table 4, and Fig. 2.

**Table 4:** Dispatch decision tree by depth, model, misclassification costs and Over/under triage rates*

Depth	Model	Misclassification costs	Over triage (%)	Under triage (%)
1	A	1:1	85.2%	0.0%
3	B	1:1	36.0%	14.1%
3	B	1:2	52.4%	13.0%
3	B	1:3 to 1:4	66.0%	10.6%
3	B	1:5	71.9%	9.4%
3	B	1:6	73.8%	8.9%
3	B	1:7	75.6%	8.5%
3	B	1:8	76.9%	8.2%
3	B	1:9 to 1:12	82.3%	6.8%
3	B	1:13 to 1:18	84.7%	4.8%
3	B	1:19	85.2%	0.0%
3	C	1:1	40.5%	14.0%
3	C	1:2	53.5%	13.0%
3	C	1:3	64.9%	11.3%
3	C	1:4	66.3%	11.0%
3	C	1:5 TO 1:8	72.7%	10.1%
3	C	1:9	85.2%	0.0%
Unlimited (7)	D	1:1	35.4%	13.8%
Unlimited (7)	D	1:2	51.6%	12.7%
Unlimited (7)	D	1:3	61.7%	11.4%
Unlimited (7)	D	1:4	67.7%	9.9%
Unlimited (7)	D	1:5	70.7%	9.3%
Unlimited (7)	D	1:6	72.0%	9.0%
Unlimited (7)	D	1:7	76.2%	8.0%
Unlimited (7)	D	1:8	78.0%	7.4%
Unlimited (7)	D	1:9	78.6%	7.2%
Unlimited (7)	D	1:10 to 1:12	80.0%	6.7%
Unlimited (7)	D	1:13	82.6%	6.1%
**Unlimited** (7)	**D**	**1:21**	**84.8%**	**2.7%**
Unlimited (7)	D	1:22	85.2%	0.0%
Unlimited (7)	E	1:1	42.1%	13.6%
Unlimited (7)	E	1:2	52.5%	12.5%
Unlimited (7)	E	1:3	56.7%	12.1%
Unlimited (7)	E	1:4	69.8%	9.9%
Unlimited (7)	E	1:5 to 1:6	70.5%	9.7%
Unlimited (7)	E	1:7 to 1:8	73.5%	9.4%
Unlimited (7)	E	1:9	83.4%	7.8%
Unlimited (7)	E	1:10 to 1:11	83.5%	7.7%
Unlimited (7)	E	1:12	85.2%	0.0%

^*^Model A variables were: MPDS dispatch codes

Model B variables were: MPDS dispatch codes, anyone trapped, vulnerable road user, airbag deployed, atmosphere, road surface

Model C variables were anyone trapped, vulnerable road user, anyone not ambulant, atmosphere, accident type

Model D variables were: MPDS dispatch codes, anyone trapped, vulnerable road user, anyone aged ≥ 75 years, day of the week, single v. multi-vehicle, airbag deployed, atmosphere, road surface, lighting, accident type

Model E variables were: MPDS dispatch codes, anyone trapped, vulnerable road user, anyone aged ≥ 75 years, day of the week, single v. multi-vehicle, airbag deployed, atmosphere, road surface, lighting, accident type

CHAID decision tree models had over triage rates that ranged from 35.4% in a decision tree with 3 levels using splits based on MPDS dispatch codes, anyone trapped, vulnerable road user, airbag deployed, atmosphere, and road surface; to 85.2% for multiple decision trees based on different combinations of MPDS dispatch and crash characteristics. See Table 4 and Fig. 2.

**Fig. 3:**
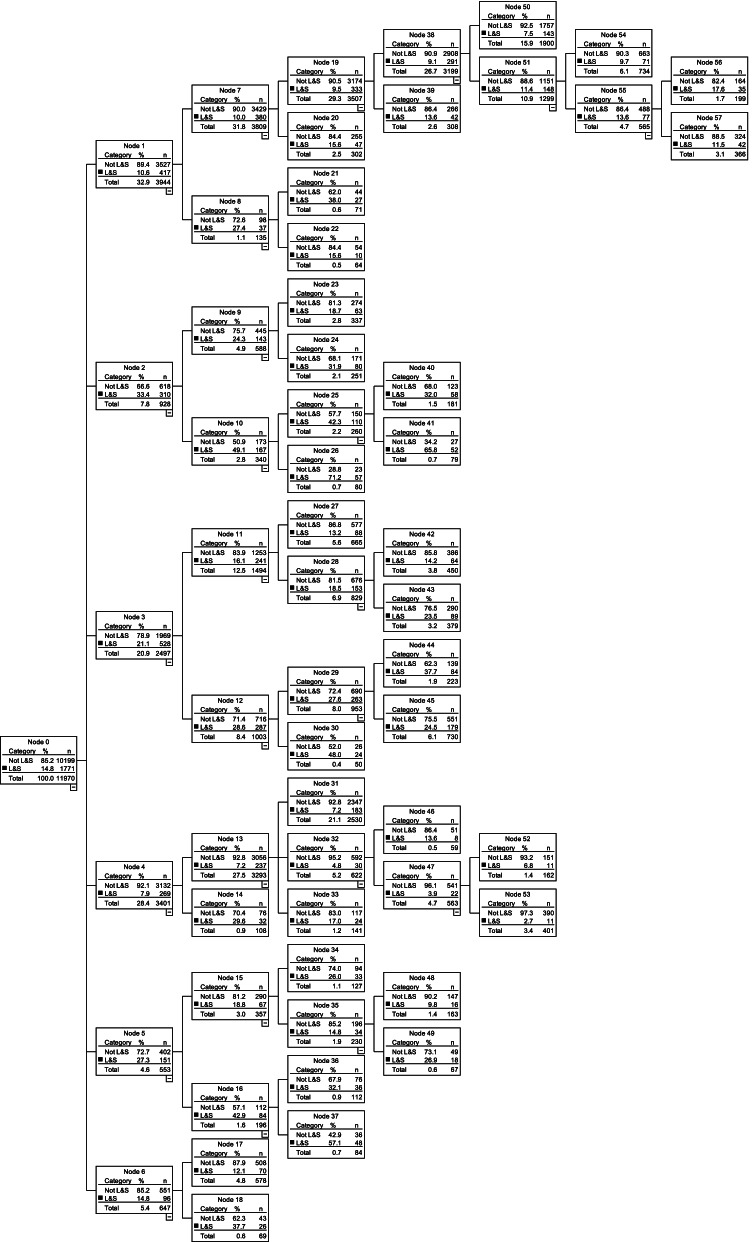
CHAID decision tree model with 84.8% over triage and 2.7% under triage rate

Figure 3 shows the decision tree that had under/over triage rates closest to the maximums proposed by the ACS COT [15], with 2.7% under triage and 84.8% over triage. This model had seven levels, with 58 nodes and 32 terminal nodes (Fig. 3).

## Discussion

A CHAID decision tree technique was used to explore different crash characteristics that had the potential to identify those MVCs that required a L&S ambulance response to the scene of a crash. Several decision trees predicted the need for a L&S response. A decision tree that would require one to ten questions asked by the EMS dispatcher of the layperson at the scene was able to predict the need for a L&S response with the lowest under triage rate. The first level of this tree was the MPDS dispatch code, followed by a combination of whether anyone was trapped, a vulnerable road user was involved, anyone was aged 75 or over, the day of the week, whether the crash involved a single or multiple vehicles, airbag deployment, the atmosphere, road surface, lighting and type of accident.

Our model had an under triage rate of 2.7% and an over triage rate of 84.8%. While not specifically for a prehospital setting, the ACS COT suggests a 5% under triage and a 25% to 35% over triage rate, as acceptable for trauma team activation at any emergency department [13]. However, EMSs are said to be ‘front loaded’, where a low under triage rate is prioritised higher than that of over triage rates [6]. A review of triage accuracy of dispatch systems for trauma patients found under triage rates varied from 1.1% to 68.0% and over triage from 4.7% to 98.8% [17]. Therefore, while this model does not reach the over triage rate set by the ACS COT, when compared to other prehospital trauma triage systems, it is within the range of what is commonly present. Additionally, it is interesting to note that this model shares some crash characteristics with the findings of others who produced similar models [8, 9, 18]. For example, a model where all patients are ambulatory, multiple vehicles involved and on a highway/interstate was able to predict the need for a L&S response to MVCs [18]. Due to differences related to ambulance availability, demand and road conditions, each EMS must define their acceptable level of risk when deciding the prioritization of ambulances [19] and may deem the under/over triage rates presented here as operationally acceptable. The importance of this variation between each EMS is aptly captured in the notion that “if you have seen one EMS, you have seen one EMS” [20].

Advanced automatic collision notification systems (AACNS) that involve in-vehicle sensors and geographic locating are progressively being included in vehicle designs [21]. AACNS provide a promising future in reducing MVC morbidity and mortality due to improved identification of injury severity and reduced response time [22]. These systems use indicators such as intrusion depth, change in velocity at impact and restraint use, to automatically relay information to EMSs regarding the predicted injury severity of the involved in the crash. These AACNS have been found to predict injury severity with under triage rates of between 5 to 13% [23]. While the number of vehicles fitted with AACNS has been limited to luxury vehicles, legislative changes, such as the eCall in Europe from 2018 [21], will provide the opportunity for EMS to improve their triage accuracy to MVCs.

Another avenue to explore improving the accuracy of identifying those MVCs that require a L&S response is through the use of machine learning algorithms. Machine learning algorithms, such as Random forest or XGBoost, are similar to the decision tree methods proposed here, although they differ in their complexity. The decision trees in this paper has a maximum depth of 7, whereas a Random forest may represent several decision trees (a forest of decision trees). While using such machine learning algorithms means the algorithms become uninterpretable, there is the potential to improve accuracy. For example, there has been some success in predicting anatomical injury (the Injury Severity Score) using crash characteristics and a machine learning methods [24]. This approach could similarly be used to predict the need or a L&S response.

### Limitations

There currently exists no standard method of retrospectively classifying those patients who required a L & S response in MVC. Our study used a composite index based on whether anyone died on scene or in transit, the priority from the scene and any medication/interventions/observations considered as requiring a L&S response. Future research could assess the utility of this measure. However, a similar method for identifying L&S has undergone preliminary validation, where detailed clinical profiling compared those who did/did not require a L&S response using a similar indicator [25]. Likewise, there exists no standard metric for EMS accuracy. Although we have used the ACS COT model, which was designed for trauma patients in a pre-hospital setting, the model was not specifically derived for decisions about EMS dispatching ambulances. It is possible that our results would have been different were a specific measure for dispatch accuracy available [16].

## Conclusions

We conclude that we were able to derive a model with a reasonable under triage rate, however, this model had an associated high over triage rate. Individual EMS, when considering their own level of risk in the prioritization of ambulances, may apply the findings here to their own jurisdictions when dispatching to the scene of a MVC. It is anticipated that the implementation of future technologies such as AACNS will improve the accuracy of identification of those MVCs that require a L&S response.

## Supplementary Information

**Additional file 1:** **Supplementary material.** List of interventions/observations/medications representing the need for alights & sirens ambulance response 

## Data Availability

The datasets generated during and/or analysed during the current study are not publicly available due to patient confidentiality. Deidentified datasets may be available after seeking relevant ethical/administrative approval from the data custodians.

## Abbreviations

AACNS: Advanced automatic collision notification systems
ACS COT: American College of Surgeons Committee on Trauma
CAD: Computer aided dispatch
CHAID: Chi-square Automatic Interaction Detector
ePCR: Electronic patient card record
EMS: Emergency medical system
HAZMAT: Hazardous materials
L&S: Lights and sirens
MRWA: Main Roads Western Australia
MPDS: Medical Priority Dispatch System
MVC: Motor vehicle crash
SJ-WA: St John Western Australia
