# Supplementary material for: A predictive ambulance dispatch algorithm to the scene of a motor vehicle crash: the search for optimal over and under triage rates
Source: BMC Emerg Med. 2022 May 6;22:74. doi: 10.1186/s12873-022-00609-5 (PMC9074212; doi:10.1186/s12873-022-00609-5)
Supplement: Supplementary file 1 — Additional file 1: Supplementary material. List of interventions/observations/medications representing the need for alights & sirens ambulance response [file 12873_2022_609_MOESM1_ESM.docx]

| **Supplementary material: List of interventions/observations/medications representing the need for a lights & sirens ambulance response** | |
| --- | --- |
| Category | Description |
| Pre-Ambulance Care | Ventilation Only |
| Pre-Ambulance Care | Cardiopulmonary Resuscitation (CPR) |
| Pre-Ambulance Care | Automated External Defibrillator (AED) - Shock delivered |
| Collapse | Ambulance Officer Witnessed |
| Collapse | Bystander Witnessed |
| Conscious State | Pain Response |
| Conscious State | Nil Response |
| Glasgow Coma Scale (GCS) Verbal | 1 None |
| Glasgow Coma Scale (GCS) Verbal | 2 Incomprehensible |
| Glasgow Coma Scale (GCS) Motor | 1 None |
| Glasgow Coma Scale (GCS) Motor | 2 Extension to Pain |
| Glasgow Coma Scale (GCS) Motor | 3 Flexion to Pain |
| Paediatric GCS Eye Opening | 1 None |
| Paediatric GCS Eye Opening | 2 To Pain |
| Paediatric GCS Verbal Response | 2 Inconsolable, Agitated |
| Paediatric GCS Motor Response | 2 Extension to Pain |
| Paediatric Motor Response | 3 Abnormal Flexion to Pain |
| Glasgow Coma Scale (GCS) Total | Total <10 |
| Head Gaze/Deviation | Present |
| Electrocardiogram (ECG/EKG) | Asystole |
| Electrocardiogram (ECG/EKG) | Bradycardia |
| Electrocardiogram (ECG/EKG) | Ventricular Tachycardia (VT) |
| Electrocardiogram (ECG/EKG) | Ventricular Fibrillation (VF) |
| Electrocardiogram (ECG/EKG) | Pulseless Electrical Activity (PEA) |
| Burns | Full Thickness |
| Burns | Airway |
| Bleeding | External considered > 500mls |
| Bleeding | Internal |
| Splint/Dressing | Traction Splint |
| Doctor at Scene | Intubated |
| E.C.G. Rhythm | Supraventricular Tachycardia (SVT) |
| Clinical Interventions | Mechanical CPR Device |
| Clinical Interventions | ST-Elevation Myocardial Infarction (STEMI) |
| Clinical Interventions | Stroke Centre Delivery |
| Breathing | Nil |
| Breathing | Shallow |
| Breathing | Slow |
| Breathing | Laboured |
| Breathing | Accessory Muscle Use |
| Breathing | Audible Wheeze |
| Splint/Dressing | Combat Application Tourniquet (CAT) |
| Airway | At-Risk/Unprotected |
| Airway | Soiled |
| Airway | Partial Obstruction |
| Airway | Complete Obstruction |
| Airway | Stridor |
| Skin Colour | Cyanotic |
| Capillary Refill | > 2 Seconds |
| Pulse | Nil |
| Pulse | Weak |
| Post cardiac arrest | Return of Spontaneous Circulation (ROSC) |
| Post cardiac arrest | ROSC Temporary |
| Post defibrillation | No Rhythm Change |
| Post defibrillation | Rhythm Change |
| Medications-Intervention | epinephrine |
| Medications-Intervention | amiodarone |
| Medications-Intervention | atropine sulphate |
| Medications-Intervention | cefazolin |
| Medications-Intervention | glucose 10% |
| Medications-Intervention | heparin Sodium |
| Medications-Intervention | metaraminol tartrate (aramine) |
| Medications-Intervention | morphine & midazolam infusion |
| Medications-Intervention | packed red blood cells |
| Medications-Intervention | rocuronium bromide (esmeron) |
| Medications-Intervention | suxamethonium Chloride |
| Medications-Intervention | tranexamic acid (TXA) |
| Skills | Needle Thoracentesis |
| Skills | Cardiopulmonary Resuscitation (CPR) |
| Skills | Cricothyrotomy |
| Skills | Defibrillator |
| Skills | Endotracheal Tube |
| Skills | Finger Thoracostomy |
| Skills | I-Gel Supraglottic Airway Device |
| Skills | Intraosseous Cannulation |
| Skills | Laryngeal Mask Airway |
| Skills | Magill Forceps |
| Skills | Oropharyngeal Airway |
| Skills | External Cardiac Pacing |
| Skills | Rapid Sequence Induction |
| Skills | Suction (of the airway) |
| Skills | Synchronised Cardioversion |
| Skills | Ventilator |
| Other finding | Amputation |
| Other finding | Partial Amputation |
